# Immunosenescence: A Critical Factor Associated With Organ Injury After Sepsis

**DOI:** 10.3389/fimmu.2022.917293

**Published:** 2022-07-18

**Authors:** Xuan Lu, Yun-Mei Yang, Yuan-Qiang Lu

**Affiliations:** ^1^ Department of Geriatric and Emergency Medicine, The First Affiliated Hospital, School of Medicine, Zhejiang University, Hangzhou, China; ^2^ The Key Laboratory for Diagnosis and Treatment of Aging and Physic-chemical Injury Diseases of Zhejiang Province, Hangzhou, China

**Keywords:** sepsis, immune aging, immunosenescence, immunosuppression, myeloid-derived suppressor cells

## Abstract

Progressive immune dysfunction associated with aging is known as immunosenescence. The age-related deterioration of immune function is accompanied by chronic inflammation and microenvironment changes. Immunosenescence can affect both innate and acquired immunity. Sepsis is a systemic inflammatory response that affects parenchymal organs, such as the respiratory system, cardiovascular system, liver, urinary system, and central nervous system, according to the sequential organ failure assessment (SOFA). The initial immune response is characterized by an excess release of inflammatory factors, followed by persistent immune paralysis. Moreover, immunosenescence was found to complement the severity of the immune disorder following sepsis. Furthermore, the immune characteristics associated with sepsis include lymphocytopenia, thymus degeneration, and immunosuppressive cell proliferation, which are very similar to the characteristics of immunosenescence. Therefore, an in-depth understanding of immunosenescence after sepsis and its subsequent effects on the organs may contribute to the development of promising therapeutic strategies. This paper focuses on the characteristics of immunosenescence after sepsis and rigorously analyzes the possible underlying mechanism of action. Based on several recent studies, we summarized the relationship between immunosenescence and sepsis-related organs. We believe that the association between immunosenescence and parenchymal organs might be able to explain the delayed consequences associated with sepsis.

## Introduction

Aging is a complex phenomenon characterized by the progressive loss of physical functions and numerous age-related diseases, such as neurological disorders, cardiovascular diseases, renal insufficiency, and chronic obstructive pulmonary disease (COPD) (1). Three main characteristics are associated with senescent cells: apoptotic resistance, cell proliferation arrest, and senescence-associated secretory phenotype (SASP) (2). Immunosenescence refers to the age-related deterioration of the immune system. The concept of immunosenescence was proposed as early as 1964 (3). The most prominent characteristics include reduced adaptive immunity and resistance to infection and an increased risk of autoimmune disorders (4). Additionally, immunosenescence is regulated by factors like excessive chronic inflammation and alterations in the microenvironment (5). Immunosenescence may lead to a vicious cycle of an imbalance between an impaired immune system and the indirect regulation of parenchymal tissues and organs (6).

Sepsis is an intricate, heterogeneous, and highly fatal syndrome (7), which is responsible for life-threatening organ dysfunction due to the immune regulation disorder. The third international consensus definition of sepsis and septic shock (Sepsis 3.0) recommended the sequential organ failure assessment (SOFA) to assess sepsis and hence predict the subsequent prognosis (8). While the old definitions of sepsis greatly emphasized infection, Sepsis 3.0 focused on the dysregulation of the body’s response to infection and organ dysfunction. Furthermore, the organ damage scored by SOFA focuses on organs like lungs, heart, liver, kidneys, and brain. Surviving sepsis is associated with chronic, long-term consequences in host protective immunity (9). Additionally, researchers observed that most of the survivors suffered from issues like nervous system disturbances and cognitive dysfunction throughout their life span (10). Since several similarities are found between immunosuppression after sepsis and immunosenescence, researchers hypothesized that these two factors might be associated with the progressive failure of immune functions (11, 12). In sepsis, an increase in the number of myeloid-derived suppressor cells (MDSCs) was associated with the regulation of the function of other immune cells, and excessive inflammation was blocked (13, 14). It has been suggested that MDSCs play a paradoxical role in sepsis: these cells may increase the production of proinflammatory cytokine during emergency myelogenesis and be also potently immunosuppressive (15). MDSCs may induce immunosenescence in the remodeled immune system (16). Therefore, we were interested in analyzing the effect of immunosenescence on sepsis, including the effect on the parenchymal organs. Here we review the possible relationship between septic injury-related organs and immunosenescence and analyze the possible mechanisms of immunosenescence after sepsis, which may shed some light on the delayed consequences of sepsis.

## Senescence in Immune Cells

**Figure 1:**
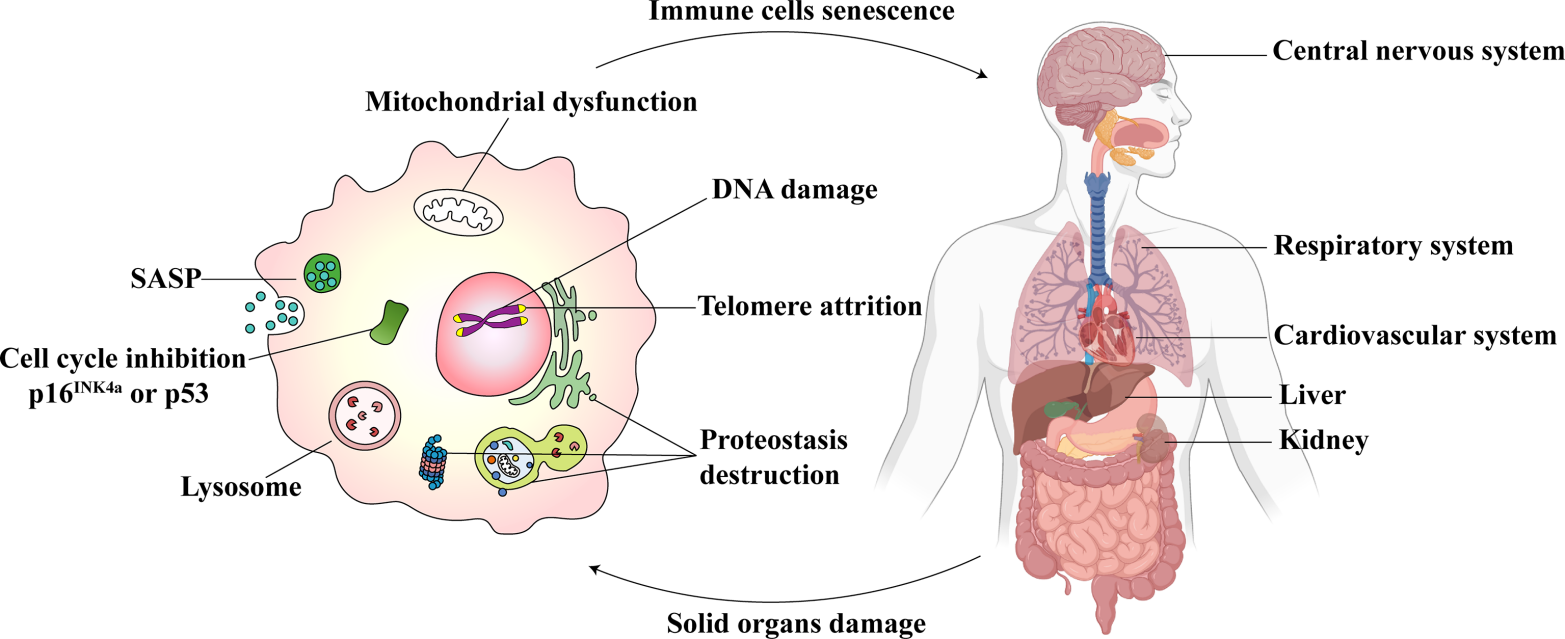
Immunosenescence and parenchymal organ damage complement each other. The senescent immune cells (Left) show excessive oxidative stress in the mitochondria. The dysfunction, in turn, leads to DNA damage, telomere attrition, and proteostasis destruction. The cell cycle is inhibited. The senescence-associated secretory phenotype (SASP) is released, and lysosomal SA-β-gal accumulates. Immunosenescence could influence the central nervous system, respiratory system, cardiovascular system, liver, and kidneys (Right). At the same time, the damage to these parenchymal organs may also cause immune senescence.

The pathogenesis associated with immunosenescence is multifactorial, which affects both innate and adaptive immunity. Immunosenescence involves the generation, migration, proliferation, differentiation, and activation stages of immune cells (16). The life-threatening consequences include a declined response to antigen stimulation, an increased response to chronic inflammation, a reduction in the defense to pathogenic microbial invasion, and a decrease in gene mutation identification and clearance ability in cells (16). These could increase the risk and severity of infectious diseases, autoimmune diseases, and cancer (17). At the cellular level, the senescence of immune cells includes atrophy and failure of normal cellular functions. The prominent features associated with the detection of senescence in immune cells are shown in **Figure 1**. They include the following: 1) Cell cycle inhibition: downstream signaling of p53/p21CIP and p16INK4a/pRB pathways and inhibition of cyclin-dependent kinases (18). 2) Telomere attrition: the shortening of telomere length, wherein the telomerase activity is impaired (19). 3) SASP: it refers to the secretion of a wide range of soluble factors by senescent cells, such as chemokines, pro-inflammatory cytokines, growth factors, and proteases (20). 4) An excess accumulation of beta-galactosidase (SA-β-gal) in the lysosomes results in pH changes in lysosomes (21). 5) Mitochondrial dysfunction: Different immune cells express different mitochondrial phenotypes after aging and are affected by oxidative stress (22). 6) DNA damage: The persistence of DNA damage can lead to apoptosis or senescence (23). 7) Proteostasis: a gradual loss of unfolded protein response with aging, which results in proteostasis (24).

## Immunosuppression of Sepsis Accelerates the Development of Immunosenescence

**Figure 2:**
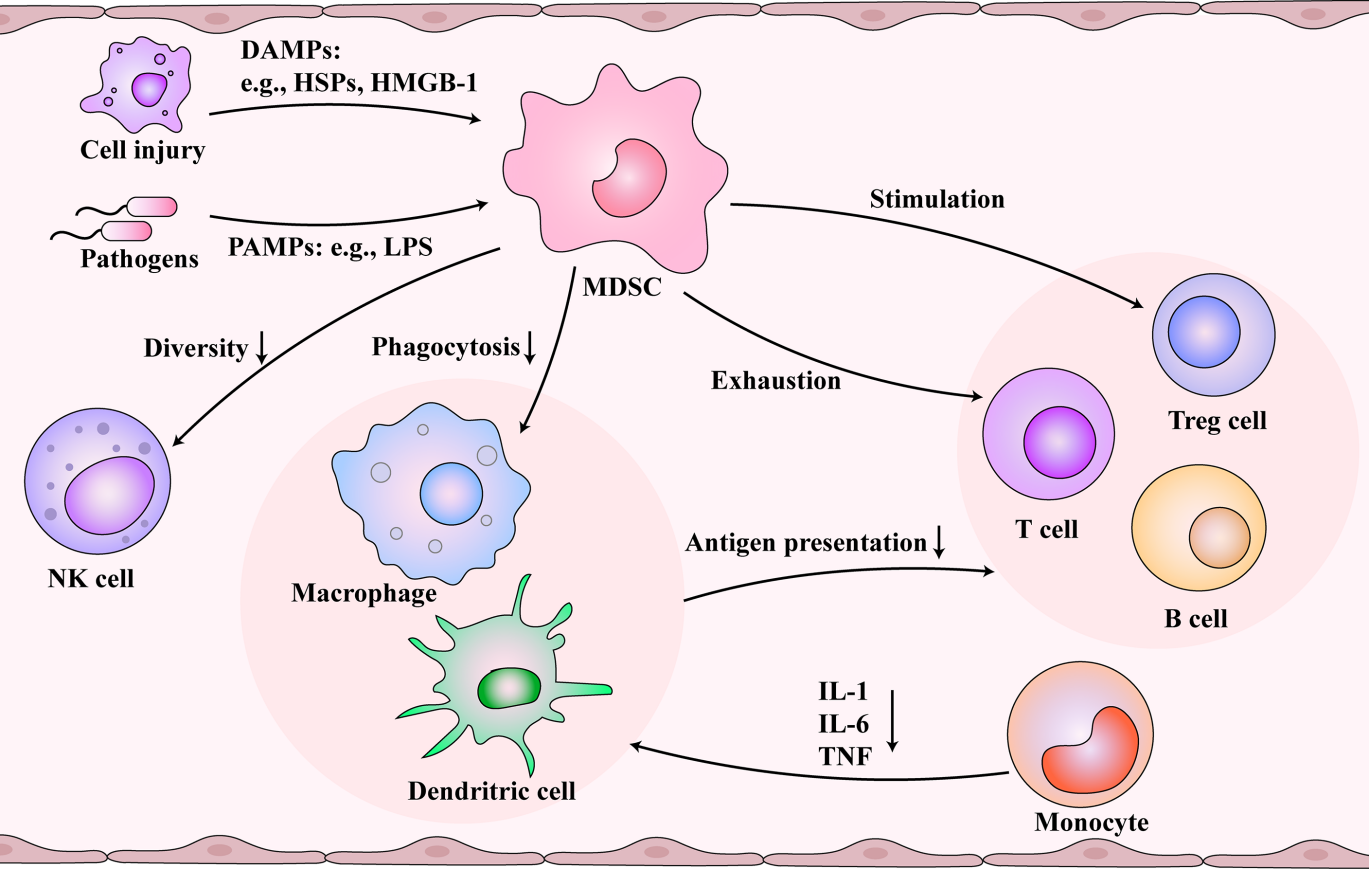
Immunosuppression and immunosenescence caused by myeloid-derived suppressor cells (MDSCs) after sepsis. Damaged cells and pathogens release damage-associated molecular patterns (DAMPs) and pathogen-associated molecular patterns (PAMPs), respectively, which are picked up by the immune system. During immunosuppression and immunosenescence stage of sepsis, MDSCs are activated and inhibit the function of dendritic cells (DCs) and macrophages. At the same time, MDSCs reduce the diversity of NK cells. Monocytes differentiate into DCs and macrophages, but MDSCs change the cytokines secreted by them. The functions of the T cells and B cells are inhibited by damaged antigen-presenting cells, controlled by MDSCs, and regulated by regulatory T (Treg) cells. Immunosenescence and immunosuppression form a vicious cycle.

Sepsis could lead to an unbalanced immune response. According to Sepsis 3.0, this phenomenon is associated with excessive inflammation and immunosuppression in the body (8). A persistent immune stimulation is characterized by invading pathogens and release of damage-associated molecular patterns (DAMPs). DAMPs activate pattern recognition receptors, which can detect the pathogen-associated molecular patterns (PAMPs), triggering a vicious cycle of continuous activation and dysfunction of the immune system (25) (**Figure 2**). In the initial stage, many pro-inflammatory factors are released, such as TNF-α, IL-1β, IL-6, IL-12, and IL-18. Further on, researchers observed that the patients with sepsis suffered profound immunosuppression. An altered immune response includes an intense “storm of inflammatory factors,” which act as immunosenescence accelerators (12). Immunosenescence is a dynamic process that diminishes immune system functions (26). It leads to a low-grade chronic inflammation called “inflammaging” and was found to reduce the ability to trigger an effective antibody and cellular response (27, 28). Therefore, chronic inflammation may be the result or an influencing factor of immunosenescence.

### Influence of Sepsis-Associated MDSCs and Immunosenescence in Immune Cells

MDSCs are a heterogeneous immature myeloid cell population that originates from hematopoietic stem cells and undergo the myeloid progenitor cell developmental stage in the bone marrow (29). In many pathological conditions, the accumulation of MDSCs occurs after an impaired myelopoietic maturation of granulocytes, monocytes, macrophages, and dendritic cells (30). Emergency myelogenesis in the bone marrow is triggered when peripheral inflammatory factors stimulate the secretion of colony-stimulating factors and chemokines (31). Gabrilovich et al. proposed a two-stage model that the expansion and activation of MDSCs require 1) emergency myelopoiesis drove by exogenous stimulus like infection and 2) pathological myeloid activation induced by the release of DAMPs or PAMPs from organ injury and secondary infections (32, 33). MDSCs are associated with prolonged immunosuppression in sepsis (34). Meanwhile, the number of MDSCs increases with age (35–37). Next, we investigated the immunosuppression caused by MDSCs, which may be associated with immunosenescence. MDSCs and aging demonstrate similar effects on immune cells, which might be essential for understanding immunosenescence after sepsis (**Figure 2**). MDSCs are essential for eliciting a macrophage immune response to inflammation (38). MDSCs inhibit the function of DCs and macrophages (39). The antigen presentation and phagocytosis function of macrophages in the blood are weakened with age (40). In monocytes, IL-1, IL-6, and TNF are reduced (41), and several defects in monocyte surface molecules, including CD14 and CD71, have also been observed in sepsis patients (42). Also, aging could inhibit the diversity of natural killer (NK) cells (43). MDSCs could reduce the secretion of cytokines and the cytotoxicity of NK cells (44, 45). Also, the effect of MDSCs and aging on T cells are profound. The cytotoxicity associated with CD8^+^ T cells is inhibited by reducing the number of IL-2 and INF-γ. Additionally, MDSCs promote T cell depletion, which is consistent with the decrease in the number of T cells with increasing age (46). Studies also found that the responsiveness of T cells to different external injuries decreases with age (47). In addition, MDSCs can stimulate the production of Treg cells and further inhibit the function of T cells (39, 48). In sepsis, B cells develop toward exhaustion. Both MDSCs and senescence contribute to the decline of B lymphocytes, including proliferation and antibody production (49, 50). The expression of MHC II decreases, and the secretion of IL-10 increases (51).

After sepsis, MDSCs increase the expression of arginase 1 (ARG1), which lowers the arginine concentration in the microenvironment (52). Also, MDSCs activation stimulates the expression of indoleamine-pyrrole 2,3-dioxygenase (IDO), which leads to tryptophan deficiency (53). As a result of the lack of both proteins, protein synthesis is inhibited, and T cell proliferation is affected. MDSCs also demonstrate the potential to enhance oxidative stress. The expression of NADPH oxidase 2 (NOX2) and inducible nitric oxide synthase (iNOS) is increased (54). The production of NO and ROS compounds oxidizes tyrosine residues on the T cell receptor (TCR), resulting in reduced anti-specific stimulation of T cells (55). Reactive oxygen species (ROS) may induce immunosenescence (56). In addition, MDSCs can also inhibit T cell function through the PD1/PDL1 axis, resulting in immunosuppression (48).

### Mechanism of Immunosenescence After Sepsis

The mechanism of immunosenescence after sepsis is a hot research topic, and several recent studies suggested many different possibilities. Telomeres, located at the end of chromosomes, control cell aging and regulate gene expression. Telomere shortening has become a putative causative event of cellular aging. Sepsis proved to cause telomere shortening with decreased telomerase activity in both mice and humans (57). Also, telomere shortening may be related to the increased oxidative stress after sepsis (58). Previous studies showed a negative correlation between the level of oxidative stress and telomere length (59).

A significant manifestation of immunosuppression is the reprogramming of immune cells, including the epigenetic regulation of gene expression through histone modification and DNA methylation (DNAm) (60). Histone acetylation of lysine residues supports transcription, while their methylation leads to the formation of active euchromatin or silent heterochromatin (61). The methylation of histone-3 lysine-27 (H3K27) and histone-3 lysine-4 (H3K4) are associated with the inhibition and activation of transcription, respectively. Lipopolysaccharide (LPS)-tolerant macrophages show an increased tumor-suppressive histone modification of H3K9 me2 in promoter regions encoding IL-1β and TNF genes. Similar results were obtained in LPS-tolerant PBMCs in sepsis patients (62). In patients with sepsis, monocytes and macrophages can also be reprogrammed. Additionally, DNAm is linked to age (63, 64). Age-related methylation in the nuclei and mitochondria DNA is considered a senescence biomarker (65). For example, methylation in the CpG islands of gene ELOVL2 is highly age-related (66, 67). Extensive research demonstrated that DNAm could be used to explore the outcome of immunosenescence (68–70).

The regulation of immune checkpoints is essential in both immunosuppression and immunosenescence after sepsis. The programmed cell death-protein 1 (PD-1), programmed death-ligand 1 (PD-L1), and cytotoxic T lymphocyte-associated protein (CTLA-4) are currently the focus of immune checkpoint proteins. Increased expression of PD-1 is associated with an increased incidence and mortality of secondary nosocomial infection after septic shock and decreased T cell proliferation (71). *In vitro* experiments demonstrated that blocking PD-1 and PD-L1 pathways could improve lymphocyte apoptosis and regulate immune function (72). PD-L1 expression of neutrophils and monocytes and PD-1 expression of CD8^+^ T cells and NK cells resulted in impaired phagocytosis (73). The immune checkpoint inhibitor (ICI) of PD-1 could improve the survival rate in septic mice (74). Similarly, anti-CTLA-4 antibody therapy reduced sepsis-induced spleen apoptosis and improved survival (75). Although a controversy still exists about whether PD-1 antagonists can alleviate aging, many studies showed that ICIs are also meaningful for immunosenescence. A recent meta-analysis was conducted on the efficacy of ICI in older and younger patients. ICIs improved overall survival in both young and old patients with a cut-off age of 65-70 years (76). However, more than one study showed that ICI is more effective in older patients with metastatic melanoma than in younger patients (77–79). Due to the heterogeneity of different ICIs and different diseases, the research on the effect of ICIs on immunosenescence is still in progress.

**Figure 3:**
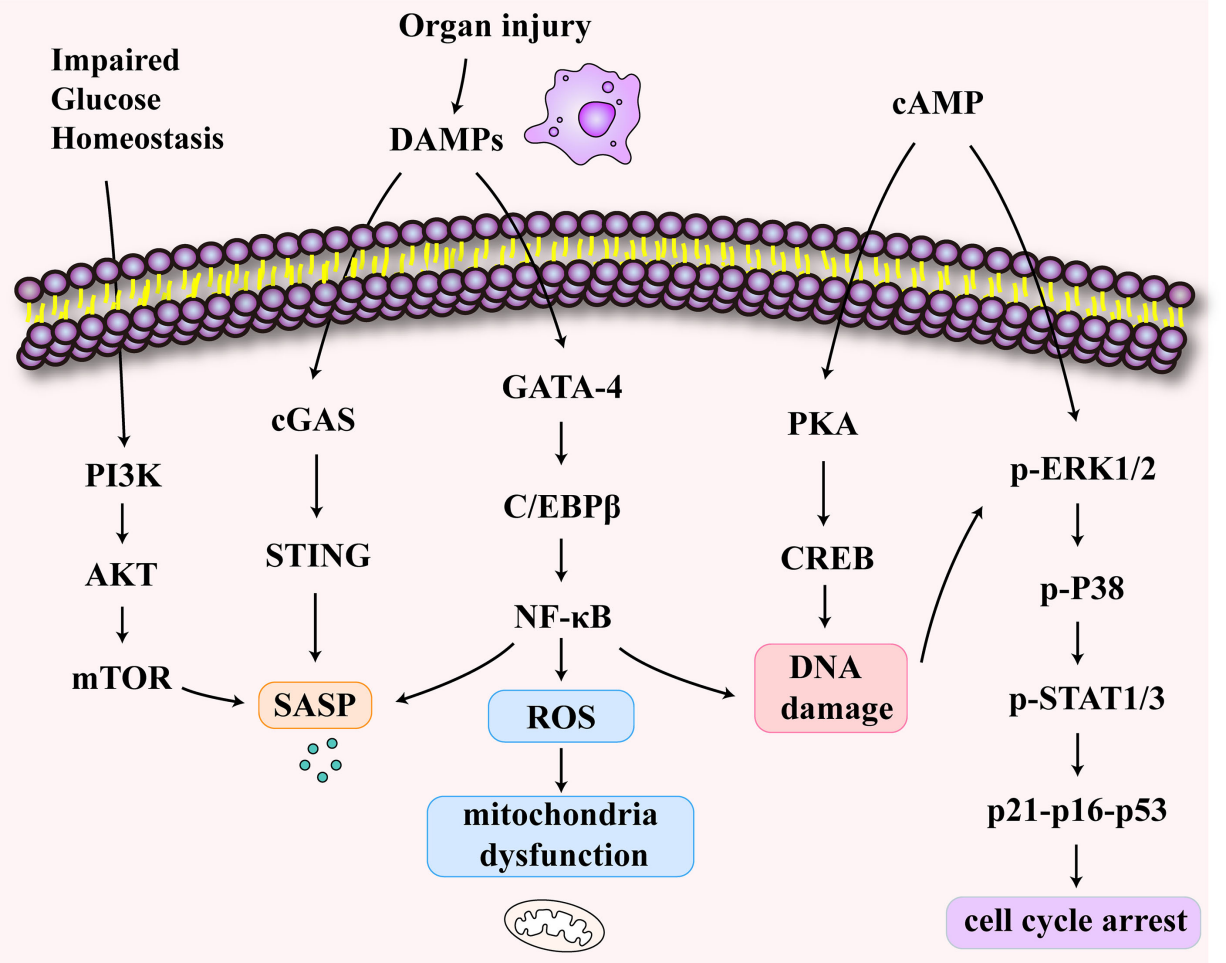
The signaling pathways of T cell senescence following sepsis-associated organ injury. The disruption of glucose metabolism activates the PI3K-AkT-mTOR signaling pathways in senescent T cells. The increase of cyclic adenosine monophosphate (cAMP) activates cAMP-PKA-CREB pathway, ERK1/2 and p38 pathways. These pathways may induce DNA damage and cell cycle arrest. The release of damage-associated molecular patterns (DAMPs) activates cGAS-STING pathway and NFκB pathway which may be related to SASP secretion and mitochondrial dysfunction.

A decline in the T cell output is a characteristic of immunosenescence (80). T cell senescence differs from anergy and depletion in that it is an irreversible process (81, 82). T cell senescence has been demonstrated in tumors and has been shown to occur in inflammaging (83, 84). Here, we explore the mechanism of T cell senescence following sepsis-associated organ injury (**Figure 3**). Impaired glucose homeostasis may occur in sepsis (85). Furthermore, the disruption of glucose metabolism activates the PI3K-AkT-mTOR signaling pathways in senescent T cells (86, 87). Studies showed that cyclic adenosine monophosphate (cAMP) production can activate the cAMP-PKA-CREB pathway, which could induce senescence in T cells (88). In addition, ERK1/2 and p38 pathways are activated by cAMP (89). The P38 arrests cell cycle progression by activating p53, p21, and p16 (90). Also, organ damage caused by sepsis produces DAMPs which activate the cGAS-STING pathway (91). At the same time, the activated NFκB pathway leads to DNA damage and SASP secretion and causes mitochondrial oxidative stress, resulting in mitochondrial dysfunction (92).

## Immunosenescence Influences Injury and Aging of Solid Organs

Immunosenescence affects immune organs, including the spleen, bone marrow, and thymus, thus accelerating the degeneration of the immune system (93). It is considered a comprehensive remodeling of the immune system and the microenvironment (94). Researchers observed that with aging, the immune system could drive systematic age-related changes in solid non-immune organs (95). This alteration can result in the loss of tissue homeostasis and cause significant damage. As immunosenescence progresses, the homeostasis of innate and adaptive immune cells in solid organs is disrupted, and the body’s susceptibility to infectious diseases and cancer increases (96). In addition to detecting aging markers, p16, p21, and SASP in the liver, kidneys, lungs, and other solid organs of immunosenescent mice, tissue damage is also observed in these organs. The aging and damage of these organs will eventually lead to a shortened lifespan. The extent of inflammation can predict the possibility of cardiovascular diseases, neurodegenerative disorders, and weakness (97). Sepsis induces damage to the organs, and growing evidence exists linking sepsis and immunosenescence. Immunosenescence may occur after sepsis and could complement organ damage (**Figure 1**). Therefore, exploring the effect of immunosenescence on solid organs is of great significance.

### Immunosenescence Increases Disease Susceptibility of Lungs

Different individualistic factors like lifestyle, long-term environmental exposure, gene factors, and systemic diseases can be used as regulatory factors of lung dysfunction, resulting in lung remodeling, impaired respiratory function, and increased susceptibility to lung diseases (98–100). When sepsis occurs, the lungs are most likely to be damaged first (101). Acute lung injury caused by sepsis, including acute respiratory distress syndrome, is one of the most common causes of death in patients with sepsis (102).

Oxidative stress-induced by immunosenescence may lead to structural and functional abnormalities in cilia (103). The activation of PKCϵ by oxidation can reduce the airway’s ciliary beat frequency and curtail the ciliary clearance function (104). The barrier function of airway epithelial cells (AECs) decreases with immunosenescence (105), and SASP secreted by senescent cells can promote senescence of AECs (106). Senescent epithelial cells can increase airway susceptibility (107). An immunological crosstalk occurs between AECs and dendritic cells (DCs). The DCs of the elderly secrete pro-inflammatory factor, TNF-α, and affects the function of primary bronchial epithelial cells, making the elderly more susceptible to chronic airway inflammation (108). DC’s activation and maturation are inhibited, and antigen presentation ability decreases in the elderly (109). In addition to its association with DCs, AECs also exhibit a synergistic effect on the airway macrophages. AECs and airway macrophages can interact to engulf the particles or pathogens (110). A study on senescent lung macrophages showed that the proliferation ability of macrophages was decreased, the production of cytokines was impaired, and the phagocytosis and killing of bacteria and the clearance effect on apoptotic neutrophils were weakened (40, 111–113).

In addition, the impaired immune function caused by immunosenescence affects the adaptive immune system of the lungs. Senescence decreases the number of B cells, transforming them from naive B cells into antigen-expressing B cells (114). Many reasons for this decrease exist, including cytokine secretion and metabolism. Studies showed that a decrease in IL-10 in old-aged mice can reduce B cells (115). Tryptophan metabolism is influenced by aging, and a decrease in tryptophan concentration inhibits B cell development (116). Thymic involution may cause T cell shifts. The distribution of immune cells might vary, and the lungs might endure pro-inflammatory metastases after immunosenescence (117). The dysfunction of T cells also includes a decrease in the number of memory and naive T cells (118). Activating thymic T-regulatory cells (Tregs) associated with Th17 and persistent chronic inflammation leads to an increase in age-related lung autoimmunity. Tregs are further activated by the release of inflammatory factors (119).

### Immunosenescence Disrupts the Blood-Brain Barrier and the Function of Microglia

Approximately 70% of sepsis patients demonstrate the possibility of sepsis-associated encephalopathy (120). Sepsis-related encephalopathy causes acute organ dysfunction and many long-term mental problems. For example, immunosenescence is associated with neurodegenerative diseases like delayed onset Alzheimer’s disease and Parkinson’s disease (PD) (121). Intestinal flora affects neural development, regulates behavior, and promotes neurological disorders (122). The location and accumulation of gut microbes are associated with immunosenescence (123). As a result, the intestinal barrier’s adhesion and leakage of microorganisms and microbial byproducts are increased (124, 125). Changes in the microbial-gut-brain axis led to an increase in circulating inflammatory factors (126). Short-chain fatty acids produced by the gut flora activate brain microglia, which cause neuroinflammation and neuron damage (127).

The blood-brain barrier (BBB) is a highly selective barrier between blood and brain tissue that limits the uncontrolled diffusion of cells and molecules from the blood into the central nervous system and regulates the entry of nutrients (128). In the case of sepsis, potentially harmful proteins can penetrate the brain through the compromised BBB (129, 130). Reduced expression of tight junction proteins inhibits endothelial cell communication, increases the BBB permeability, and decreases the BBB repair response (131). Next, P-GP and LRP-1 are essential efflux transporters that mediate amyloid-β peptide (Aβ) clearance in brain tissues (132). The expression of P-GP and LRP-1 proteins decreases with immunosenescence, leading to the accumulation of Aβ in the brain (133–135). Also, it affects the function of the glucose transporter 1 (GLUT1) on BBB vascular endothelial cells, leading to reduced glucose uptake by the brain and a reduced BBB function (136, 137). It can be speculated that immunosenescence results in increased permeability and decreased function of BBB, resulting in some potentially harmful proteins entering the brain, accumulation of Aβ, and reduced regulation of nutrient intake.

The choroid plexus (CP) is a part of the BBB, wherein almost all types of immune cells can be found, and uninterrupted crosstalk occurs through TNFα and IFNγ released by the central nervous system (138). An increase in the Th1/Th2 ratio during aging leads to an increased expression of the chemokine, CCL11, which may be associated with cognitive impairment (139). Also, an increase in CCL11 can transform the microglia into an inflammatory state (140). Thus, immunosenescence may disrupt the intrinsic balance of immune cells in the CP, leading to central nervous system dysfunction.

Microglia cells are innate immune cells in the brain that generally remain stable and in a low replication rate. Senescent microglia cells exhibit significantly smaller and less branched dendrites, exhibit slower acute responses, lower motility, and cell mobility (141). Several studies showed that aging microglia show higher proliferation and promote the production of pro-inflammatory cytokines (142). However, chemotaxis and Aβ scavenging ability attenuate, and the protective function of the central nervous system decreases. In addition, the long-term activation of microglia results in chronic inflammation of the central nervous system, which increases oxygen free radicals, mitochondrial damage, and possibly the death of neurons (142). These may further be associated with the damage of Triggering Receptor Expressed on Myeloid Cells 2-DNAX activation protein 12 (TREM2-DAP12) and CX3Cl1-CX3Cr1 axes (143). TREM2-DAP12 axes could promote microglial cell activation and phagocytosis (144). CX3Cl1-CX3Cr1 refers to an important communication channel between neurons and microglia (145). Immunosenescence disrupts both of these pathways leading to the development of neurodegenerative diseases (146). Oxidative stress-related mechanisms prior to Aβ oligomerization trigger microglia senescence and malnutrition (147). Immune receptors such as major histocompatibility complex (MHC) II, CD68, CD14, CD11, and TLRs are upregulated by aging microglial cells (148, 149). In senescent microglia and macrophages, lipid messenger prostaglandin E2 (PGE2) signaling promotes glucose conversion to glycogen through its EP2 receptor. At the same time, a decreased glucose flux and weakened mitochondrial respiration leads to maladaptive pro-inflammatory responses (150).

The phagocytosis and chemotaxis abilities of macrophages are inhibited by senescence (151). When induced by accumulated Aβ, macrophages switch from a regulatory and anti-inflammatory M2 phenotype to a pro-inflammatory M1 phenotype, producing IL-1B and TNF-α (152). Next, macrophages that interact with microglial cells and amyloid plaques are thought to be inflammatory activators that produce inflammatory cytokines and ROS, leading to neuronal loss and apoptosis (121). Therefore, immunosenescence can be manifested as the destruction of inherent barriers and the release of pro-inflammatory mediators by the interaction of microglial cells and neurons, leading to the occurrence and development of diseases.

### Immunosenescence Is Related to Cardiac Insufficiency

Heart failure and immunosenescence are mutually reinforcing processes. Chronic heart failure (CHF) is characterized by high levels of cytokines, such as IL-6, in an inflammatory state, which may also induce immune aging (153). Moreover, the more intense T cell differentiation in CHF patients, the more serious is the senescence degree (154). Additionally, the telomere length of lymphocytes is reduced by 40% in CHF (155).

Patients with sepsis may experience local myocardial ischemia or infarction secondary to coronary artery disease (156). In myocardial infarction, inflammatory signaling is a crucial reparative pathway in regulating the deposition and metabolism of extracellular matrix proteins (157, 158). Macrophage-derived matrix metalloproteinase 9 (MMP-9) can directly or indirectly improve wound healing and remodeling after myocardial infarction (159). Immunosuppression delayed granulation tissue formation and decreased collagen deposition after myocardial infarction in elderly mice to prevent myocardial remodeling (160). Studies showed that T cell metabolic failure caused by abnormal mitochondrial function could also serve as an accelerating factor of cardiovascular changes and affect organ function and life span (97). Mitochondrial transcription factor A (TFAM) deficiency could accelerate T cell senescence. Blocking of TNF-α signal transduction can partially reduce premature senescence in TFAM deficient T cells (97). Transplantation of senescent T cells into immune-deficient mice accelerated angiotensin (Ang) II-induced cardiovascular fibrosis (161). In aging patients, reducing B lymphocytes and their subsets in peripheral blood associated with chronic cardiac insufficiency paralleled the altered expression of miRNA miR-181c involved in lymphogenesis (162). These findings suggest that immune-aging-related miRNAs might play an essential role in age-related cardiac insufficiency.

### Immunosenescence Interacts With Renal Dysfunction

Acute kidney injury (AKI) is one of the most common complications associated with sepsis in the intensive care unit (ICU) (163). However, the pathophysiological mechanism of sepsis-related to renal injury remains unclear. Innate immune cells of the kidney and white blood cells in the peripheral blood circulation are associated with AKI and chronic kidney disease (CKD) (164, 165). In other words, CKD can lead to accelerated aging of the immune system and increase AKI risk (166) Analysis proved that the loss of renal function is related to the dysregulation of circulating T cells. Thymus output was significantly reduced in patients with end-stage renal disease (ESRD), and the susceptibility of naive T cells to apoptosis was increased, equivalent to 20 to 30 years of T cell aging (167). T cells also demonstrate a highly variable maturation phenotype in children with kidney disease, especially in patients who have been treated with immunosuppressive drugs (168). This suggests that a uremic environment may lead to premature senescence of T cells, which is manifested by the decrease of naive T cells and the increase of CD8^+^ T_EMRA_ cells and pro-inflammatory monocytes (169). Also, similar immune changes were positively correlated with the duration of renal dialysis (170). In a multi-ethnic population-based cohort study, an algorithm for predicting aging based on DNA methylation percentage (DNAm-based age) confirmed that immunosenescence was an essential factor influencing kidney disease (171). Senescent T cells may lead to renal fibrosis and promote the expression of inflammatory factors and the production of superoxide in the kidneys. The senescent T cells could stimulate inflammatory cytokine expression and oxidative stress in Ang II-treated renal epithelial cells (161). Age-dependent tertiary lymphoid tissue formation can promote intra-renal inflammation (172). Researchers confirmed that CD153 and CD30 signaling may interact with CD153^+^PD-1^+^CD4^+^ senescence-associated T cells (SAT) and CD30^+^ T-bet^+^ age-related B cells, resulting in tertiary lymphoid tissue generation in chronically inflammatory organs, thereby promoting renal inflammation and fibrosis (173). In addition to ESRD, CD8^+^ T cell depletion occurs as early as CKD3 in diabetes mellitus, showing the characteristics of aggravated immunosenescence (174). Moreover, the occurrence and development of kidney diseases might be associated with cardiovascular diseases. Clinical studies demonstrated that telomere shortening of leukocytes in patients with CHF may be associated with renal dysfunction (175).

### Immunosenescence and Liver: An Extensive Exploration Is Required

Liver dysfunction after sepsis can be a risk factor for multiple organ dysfunction and death caused by sepsis. The liver acts as an immune organ. In sepsis, the liver-mediated immune response can be responsible for removing bacteria and toxins but can also lead to inflammation, immunosuppression, and organ dysfunction (176). Also, liver damage may be present in the early stages of sepsis (177). However, only a few studies are found on immunosenescence during liver injury, and immunosenescence has been found in several liver diseases. The mean telomere length of white blood cells was shorter in patients with cirrhosis than in controls (178). In liver transplant recipients, lymphocytes expressed more mature phenotypic markers than controls. Also, the telomeres of lymphocytes became shorter (179). Several innate immune cells in the liver, such as resident macrophages, known as Kupffer cells, are the most representative immune cells in the liver (180). As the liver ages, pro-inflammatory cells accumulate, and the secretion of IL-6 by Kupffer cells increases (181). Some studies showed that the telomere length of Kupffer cells decreased while the number increased, and the phagocytosis ability was enhanced (182). Very few studies exist on the influence of aging on liver immunity, and further studies are needed.

## Conclusion

As a manifestation of immune dysfunction, immunosenescence plays a critical role in the occurrence and development of sepsis and affects the function of solid organs. We reviewed the current research on the effects of immune aging on septic-related solid organs. We found that immunosenescence and organ damage were mutually reinforcing processes that are linked and promote each other. At the same time, we summarized the role of MDSCs in sepsis and immunosenescence and analyzed its regulatory mechanisms. These may contribute to delayed outcomes after sepsis. The suggestion of immunosenescence in sepsis exhibits similarities to the immunosuppression stage of sepsis. However, the relationship between immunosenescence and sepsis is still undefined, and more evidence is required to confirm it.

## Author Contributions

XL conceived and completed the manuscript. Y-QL and Y-MY supervised and revised the manuscript. All authors contributed to the article and approved the submitted version.

## Conflict of Interest

The authors declare that the research was conducted in the absence of any commercial or financial relationships that could be construed as a potential conflict of interest.

## Publisher’s Note

All claims expressed in this article are solely those of the authors and do not necessarily represent those of their affiliated organizations, or those of the publisher, the editors and the reviewers. Any product that may be evaluated in this article, or claim that may be made by its manufacturer, is not guaranteed or endorsed by the publisher.
